# Genome-wide identification and expression profile of *GhGRF* gene family in *Gossypium hirsutum L.*

**DOI:** 10.7717/peerj.13372

**Published:** 2022-05-13

**Authors:** Kun Liu, Nosheen Kabir, Zhenzhen Wei, Zhuojing Sun, Jian Wang, Jing Qi, Miaoyang Liu, Ji Liu, Kehai Zhou

**Affiliations:** 1 Henan Key Laboratory of Crop Molecular Breeding and Bioreactor, Key Laboratory of Plant Genetics and Molecular Breeding, Zhoukou Normal University, Zhoukou, Henan, China; 2 State Key Laboratory of Cotton Biology, Institute of Cotton Research, Chinese Academy of Agricultural Sciences, Anyang, Henan, China; 3 Development Center for Science and Technology, Ministry of Agriculture and Rural Affairs, Beijing, China

**Keywords:** *Gossypium hirsutum*, Growth regulating factor, MicroRNA396, Gene editing, Molecular improvement, Collinearity, Expression pattern, Transcripts, Sequence logos, Gene structure

## Abstract

**Background:**

Cotton is the primary source of renewable natural fiber in the textile industry and an important biodiesel crop. Growth regulating factors (*GRFs*) are involved in regulating plant growth and development.

**Methods:**

Using genome-wide analysis, we identified 35 *GRF* genes in *Gossypium hirsutum*.

**Results:**

Chromosomal location information revealed an uneven distribution of GhGRF genes, with maximum genes on chromosomes A02, A05, and A12 from the At sub-genome and their corresponding D05 and D12 from the Dt sub-genome. In the phylogenetic tree, 35 *GRF* genes were divided into five groups, including G1, G2, G3, G4, and G5. The majority of *GhGRF* genes have two to three introns and three to four exons, and their deduced proteins contained conserved QLQ and WRC domains in the N-terminal end of *GRFs* in *Arabidopsis* and rice. Sequence logos revealed that *GRF* genes were highly conserved during the long-term evolutionary process. The CDS of the *GhGRF* gene can complement MiRNA396a. Moreover, most *GhGRF* genes transcripts developed high levels of ovules and fibers. Analyses of promoter cis-elements and expression patterns indicated that GhGRF genes play an essential role in regulating plant growth and development by coordinating the internal and external environment and multiple hormone signaling pathways. Our analysis indicated that *GhGRFs* are ideal target genes with significant potential for improving the molecular structure of cotton.

## Introduction

Cotton (*Gossypium* spp.) is the most important natural fiber and biodiesel crop in the world (Eevera & Pazhanichamy, 2013; Li et al., 2015; Zhu & Li, 2013). However, the yield and quality of cotton production must be improved. Cotton fiber and oil are closely related to the growth and development of cotton plants, and genes involved in controlling cotton growth and development can be used to improve cotton varieties. The GRF (growth-regulating factors) gene family is a branch of the small plant-specific transcription factor (TF) gene family, whose first identified member “*OsGRF1*” was isolated by differentially displaying the mRNA from internode intercalary meristem tissues of rice treated with gibberellin (Van der Knaap, Kim & Kende, 2000). Since then, many genome-wide characterizations of *GRF* genes have been performed and genome-sequencing technology has advanced. The *GRF* family is universally present in many plant taxa. For example, nine GRF family in *Arabidopsis* (Kim, Choi & Kende, 2003), 12 in *Oryza sativa* (Choi, Kim & Kende, 2004), 14 in *Zea mays* (Zhang et al., 2008), 10 in *Brachypodium distachyon* (Fi̇li̇z, Koc & Tombuloğlu, 2014), 17 in *Brassica rapa* (Wang et al., 2014a), eight in *Vitis vinifera,* 19 in *Populus trichocarpa* (Cao et al., 2016), 10 in Fragaria vesca (Li et al., 2021), 10 in *Jatropha curcas* (Tang et al., 2021), and 25 in *Nicotiana tabacum* (Zhang et al., 2018).

Many studies assessing the function of *GRF* genes have been performed in model plants such as *Arabidopsis*, rice, maize, and oilseeds. Research has revealed that *GRF* family TFs are required to regulate plant growth and development (Horiguchi, Kim & Tsukaya, 2005; Kim, Choi & Kende, 2003; Kim & Kende, 2004; Kim & Lee, 2006; M et al., 2020; Omidbakhshfard et al., 2018; Van der Knaap, Kim & Kende, 2000; Wang et al., 2014a; Wu et al., 2014), cope with environmental stresses (Fina et al., 2017; Kim et al., 2012; Shang et al., 2018), produce the syncytium during nematode infections (Hewezi et al., 2012), and establish connections with mycorrhiza during plant root growth (Bazin et al., 2013). Over-expression of *BnGRF2* (growth-regulating factor 2-like gene of *Brassica napus*) increases seed mass and oil production by upregulating the expression of chloroplast-related genes to increase chlorophyll content and enhance photosynthetic efficiency (Liu et al., 2012). Mutations of *OsGRF4* in rice cultivars can significantly enhance grain weight and increase grain yield (Beltramino et al., 2021; Che et al., 2015; Chen et al., 2019; Duan et al., 2015; Li et al., 2016; Sun et al., 2016).

*GRF* is a plant-specific transcription factor that plays an essential role in the development of roots, stems, and leaves and in the formation of flowers and seeds. However, *GRF* expression is controlled by miRNA396, and the regulation mode of GRF-miRNA396 is a core of plant development (Noon, Hewezi & Baum, 2019; Omidbakhshfard et al., 2015). The regulatory network formed by GRF and miRNA396 is essential for developing soybean roots (Noon, Hewezi & Baum, 2019). MiRNA is a small RNA regulator, typically 20-21 nucleotides in length, that can regulate the post-transcriptional inhibition of target genes. MiRNAs are a crucial regulator during plant growth and development. Heterologous miRNA396 expression in *Arabidopsis* and tobacco led to a decrease in the expression of three of the four tested NtGRF genes, which was accompanied by a severe reduction in leaf size and narrow leaf phenotype. miRNA396 expression is positively regulated by upstream TCP transcription factors, while miRNA396 regulates the transcriptional abundance of downstream GRF. miRNA396/GRF regulatory network affects the development of plant leaves (Curaba, Singh & Bhalla, 2014; Omidbakhshfard et al., 2015). The conserved miRNA396 is involved in growth, development, and abiotic stress responses in various plants by regulating its target gene growth regulator (GRF) transcription factor genes (Noon, Hewezi & Baum, 2019).

Cotton is an essential cash crop and produces 90% of the world’s lint. Currently, 45 diploids and five tetraploid cotton species are present in the cotton genus (Huang et al., 2021; Wendel & Cronn, 2003). Allopolyploid cotton (*G. hirsutum* and *G. barbadense*) is widely cultivated because of its significant economic value. *G. hirsutum* and *G. barbadense* (A and D subgenomes) were derived about 1–2 million years ago (MYA) from isolated diploid genomes of *G. arboreum* and *G. raimondii*. These diploid cotton species were hybridized through transoceanic dispersal (Hu et al., 2019; Huang et al., 2020; Wendel & Cronn, 2003). These diploid and tetraploid species are primarily used for evolutionary and biological studies of cotton.

Advancements in cotton genome sequencing (Li et al., 2015; Li et al., 2014; Wang et al., 2012) have provided a new opportunity to exploit gene resources to genetically improve cotton. In this study, we identified *GRF* genes in tetraploid upland cotton (*G. hirsutum*), the main cultivated cotton species (Li et al., 2015). The phylogenetic tree was generated using the protein sequences of *AtGRFs*, *OsGRFs,* and *GhGRFs*. Furthermore, we analyzed the exon and intron structures of the *GhGRF* genes, reverse complementary fragments in their mRNA with microRNA396 sequences, and the deduced protein motifs, as well as the *cis*-acting regulatory elements in their promoter regions. We also characterized their expression profiles in different organs, developmental stages, and responses to various abiotic and hormonal stresses. Our results provide a foundation for further studies assessing how *GhGRF* genes can be used to improve cotton genetics.

## Materials & Methods

### Identification of *GRF* genes

The genomic and protein sequences of *G. hirsutum* (NAU, v1.1; ZJU, v 1.0; JGI, v1.1) were downloaded from CottonFGD (https://cottonfgd.org/about/download.html) (Zhu et al., 2017), NCBI (https://www.ncbi.nlm.Nih.gov/genome/), and Phytozome 13 (https://phytozome.jgi.doe.gov/pz/portal.html). *GRF* protein sequences of rice were downloaded from NCBI GenBank, and the protein sequences of *AtGRFs* were acquired from TAIR 10 (http://www.arabidopsis.org). *AtGRF* sequences were used as query sequences to blast against the *G. hirsutum* protein database to search homologous candidate sequences in the *GRF* gene family with default parameters. The candidate sequences were submitted to the Batch CD-search tool in NCBI and ScanProsite tool in the ExPASy prosite database (https://prosite.expasy.org/ scanprosite/) to detect QLQ and WRC domains, which are the structural features to distinguish *GRF* family members from other gene families. The GRF family genes were determined by predicting the conserved domain of the candidate sequence.

### Gene structure and protein motifs of *GhGRFs*

We used the online software Gene Structure Display Server (GSDS) for gene structure analysis (http://gsds.cbi.pku.edu.cn/) (Hu et al., 2015), as previously described (Wang et al., 2021). The CDS sequences were aligned with the reverse complementary sequence of miRNA396a, which was acquired from previously published research papers (Hewezi et al., 2012; Yang et al., 2009). Motif analysis of the full-length protein sequence was performed using the MEME online software (Bailey et al., 2015) (https://meme-suite.org/meme/) as previously described (Qanmber et al., 2019b), according to the following parameters: classic mode, zero or one occurrence per sequence, and motif number 10.

### Biophysical properties and sequence logos of *GhGRF* genes

Various physical and chemical parameters, such as the number of amino acids, MW (molecular weight), pI (isoelectric point), and GRAVY (grand average of hydropathicity) of *GhGRF* gene coding protein sequences were determined using the ExPASy ProtParam tool (http://us.expasy.org/tools/protpara-m.html). The GRF protein sequences were aligned using Muscle in MEGA 5.2 (Tamura et al., 2011), and the results were analyzed with the WEBLOG online program (http://weblogo.plusone.com/create.cgi) (Crooks et al., 2004) as previously described (Ali et al., 2020; Qanmber et al., 2019a) to generate the sequence logos of conserved amino acid residues.

### Classification and phylogenetic analyses of *GRF* genes

Classification and phylogenetic analyses were performed using MEGA 5.2. A neighbor-joining tree of *G. hirsutum, O. sativa,* and *Arabidopsis* was constructed using full-length protein sequences according to the following parameters: Poisson correction, 95% partial deletion, and bootstrap analysis with 1000 replicates, as previously described (Xiao et al., 2018; Yu et al., 2018). Another phylogenetic tree was built using maximum likelihood (ML) methods (default parameters and JTT+G as amino acid substitution models) to confirm the authentication of the phylogenetic tree.

### Promoter *cis*-elements and expression pattern analyses of *GhGRF* genes

For *cis*-element analysis, 2.5-kb upstream promoter regions were downloaded from CottonFGD and submitted to the plantCARE database (https://bioinformatics.psb.ugent.be/webtools/plantcare/html/) (Lescot et al., 2002), as previously described (Qanmber et al., 2019a; Wu et al., 2021). The FPKM value of *GhGRF* gene expression was downloaded from CottonFGD to draw heatmaps and demonstrate the expression profiles of *GhGRF* genes when developing ovules and fibers, mature organs (calycle, leaf, petal, pistil, root, stamen, stem, and torus), germinating seeds, cotyledons, roots, and seedlings treated with abiotic stresses and hormonal treatments.

### Plant material and Real-time quantitative PCR

For expression pattern analysis, tissue samples were collected under field conditions. Cotton plants growing under normal conditions were placed into a container with a predetermined concentration of BL, IAA, JA, PEG, and NaCl. The samples were collected at different time points (0, 0.5, 1, 3, and 5 h), immediately frozen into liquid nitrogen, and stored at −80 °C for subsequent RNA extraction and qRT-PCR analysis. We used the Trizol (Invitrogen, Carlsbad, CA, USA) method to extract total RNA from each cotton sample, as previously described. cDNA was synthesized using a PrimeScript 1st strand cDNA Synthesis Kit (Takara), according to the manufacturer’s instructions. RT-qPCR was performed using SYBR Green Master Mix (Takara) on an IQ5 Real-Time PCR Detection System (Bio-Rad, Hercules, CA, USA) (Wang et al., 2014b). RT qPCR experiments were performed using the actin gene as an internal reference gene. The PCR program consisted of an initial polymerase activation at 95 °C for 1 min, followed by 40 cycles of 95 °C for 10 s and 60 °C for 30 s. Upon completion of the PCR, a dissociation curve was generated to confirm the specificity of the product and avoid generating primer dimers.

## Results

### Identification and naming of *GhGRF* genes

**Figure 1:**
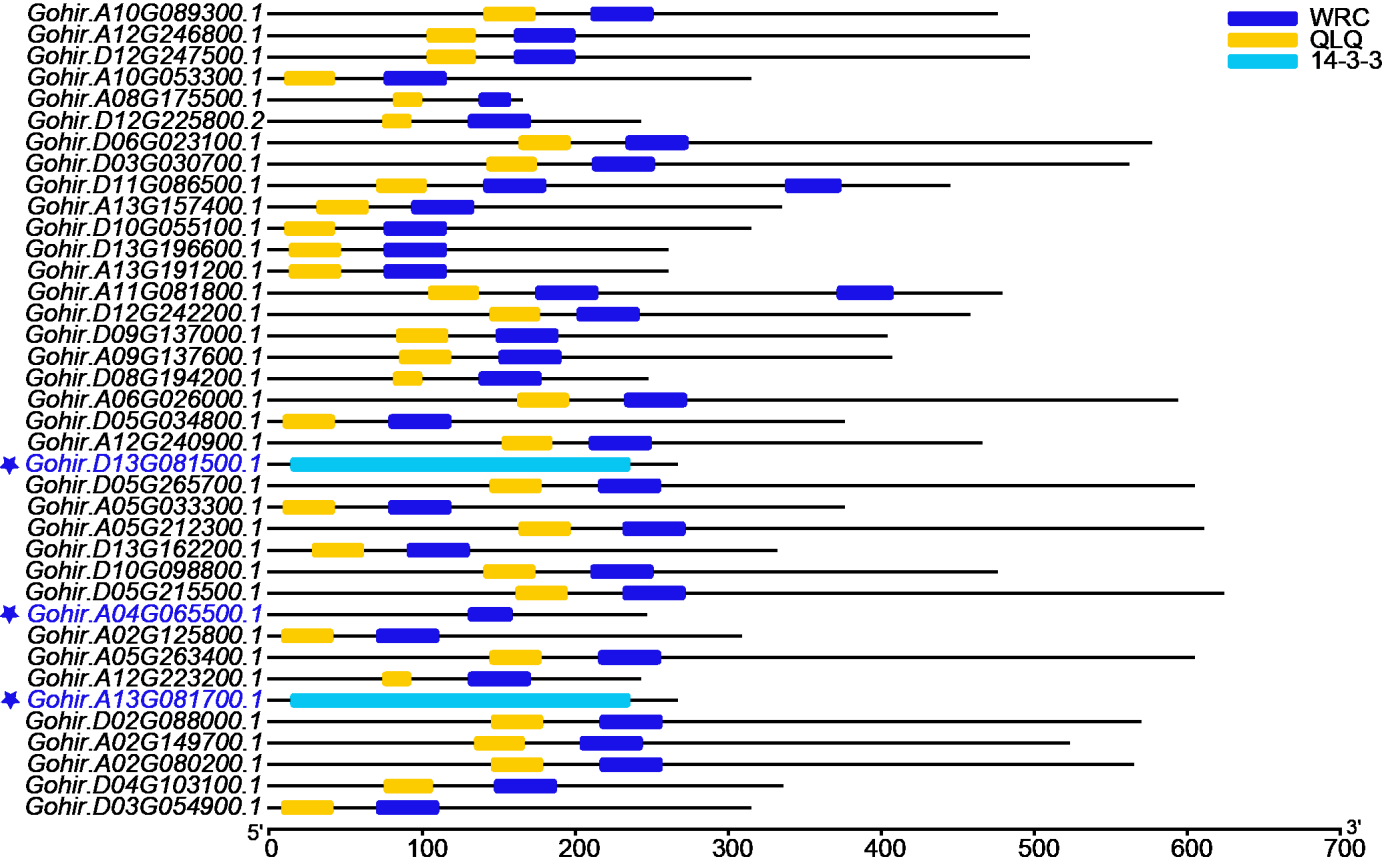
The domain analyses of 38 *GhGRF* candidates identified from JGI; 35 out of 38 protein sequences were confirmed to contain the QLQ domain adjacent to the WRC domain in the N-terminal end. The red font with preceding asterisks indicates discarded sequences.

To identify the *GRF* gene family, candidate sequences lacking both domains (QLQ and WRC) or with only one of the two domains were discarded. Finally, 35 *GRF* genes were identified (Fig. 1). To further enhance the credibility of the results, two additional blasts were performed against NAU and ZJU protein sequence data of *G. hirsutum*. The 42 or 41 candidates were also submitted to the Batch CD-search tool in NCBI, and 35 *GhGRF* family members were confirmed (Figs. S1 and S2). Multiple sequence alignment was performed using JGI (35), N AU (33), and ZJU (35) *GhGRF* deduced protein sequences. The gene names and their corresponding IDs are displayed in Table 1, and the nomenclature is described in detail in later paragraphs.

### Conserved amino acid residues within QLQ and WRC domains

**Figure 2:**
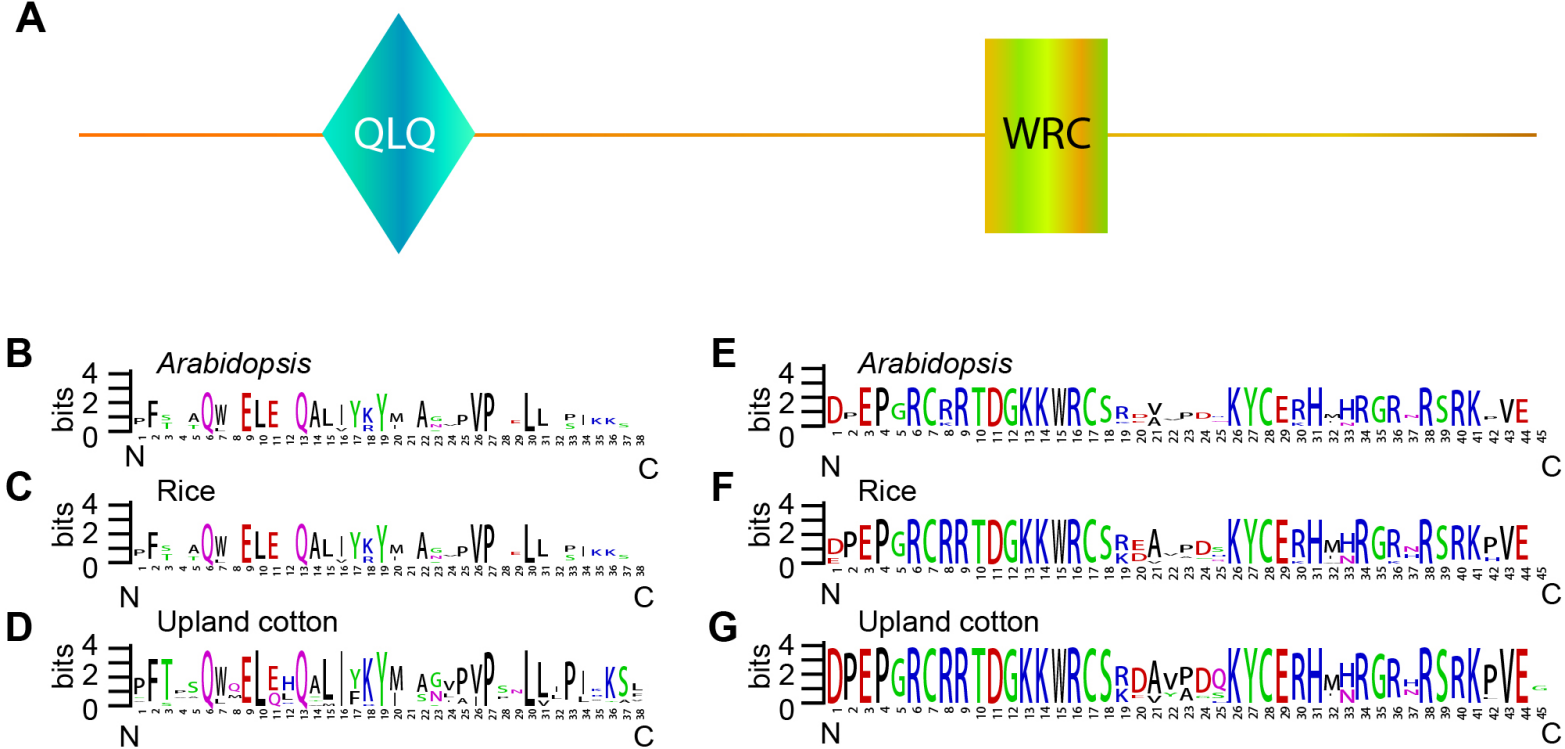
Sequence logos of *GRF* genes. Sequence logos showing the highly conserved QLQ and WRC domains (A) respectively in *Arabidopsis* (B, E), rice (C, F), and upland cotton (D, G).

The *GRF* gene family members were characterized by the presence of both conserved QLQ and WRC domains in the N-terminal end (Kim, Choi & Kende, 2003; Van der Knaap, Kim & Kende, 2000). To further explore the conservative properties of the QLQ and WRC domains of *GhGRFs*, multiple sequence alignment was performed to depict sequence logos of QLQ and WRC domains for *G. hirsutum*, *Arabidopsis,* and rice. The conserved domain sequence logos showed that most amino acid residues distributed in two domains were highly conserved among three plant species (Fig. 2). Amino acid residues include Q (6), E (9), Q (13), Y (19), V (26), P (27), and L (30) in the QLQ domain, while EP (3-4), RC (6-7), RRTDGKKWRC (9-17), KYC (26-28), H (31) and R (38) in the WRC domain were highly conserved.

**Table 1:** Names and characteristics of the *GRFs* in *G. hirsutum*. Chr means chromosome, str means the gene is on the positive and negative chain.

Gene name	ID	Chr(str)	CDS(bp)	Exon no.	Length(aa)	MW(kDa)	pI	GRAVY
GhGRF1a_At	Gohir.A06G026000.1	At06(-)	1,782	4	593	64.42	8.83	−0.458
GhGRF1a_Dt	Gohir.D06G023100.1	Dt06(-)	1,731	4	576	62.39	6.48	−0.453
GhGRF1b_At	Gohir.A02G149700.1	At02(+)	1,569	4	522	57.25	7.71	−0.612
GhGRF1b_Dt	Gohir.D03G030700.1	Dt03(-)	1,686	4	561	61.30	7.70	−0.630
GhGRF1c_At	Gohir.A05G212300.1	At05(-)	1,833	4	610	65.38	8.36	−0.482
GhGRF1c_Dt	Gohir.D05G215500.1	Dt05(-)	1,872	5	623	68.37	8.95	−0.373
GhGRF1d_At	Gohir.A10G089300.1	At10(+)	1,428	4	475	52.25	6.63	−0.566
GhGRF1d_Dt	Gohir.D10G098800.1	Dt10(-)	1,428	4	475	52.03	6.34	−0.519
GhGRF1e_At	Gohir.A05G263400.1	At05(-)	1,815	4	604	65.25	6.58	−0.577
GhGRF1e_Dt	Gohir.D05G265700.1	Dt05(-)	1,815	4	604	65.41	6.48	−0.568
GhGRF1f_At	Gohir.A02G080200.1	At02(+)	1,695	4	564	61.29	7.26	−0.558
GhGRF1f_Dt	Gohir.D02G088000.1	Dt02(+)	1,710	4	569	61.70	6.97	−0.581
GhGRF3a_At	Gohir.A09G137600.1	At09(+)	1,221	4	406	44.23	7.83	−0.684
GhGRF3a_Dt	Gohir.D09G137000.1	Dt09(+)	1,212	4	403	43.82	8.24	−0.646
GhGRF3b_Dt	Gohir.D04G103100.1	Dt04(-)	1,008	4	335	36.73	9.28	−0.518
GhGRF5a_At	Gohir.A13G191200.1	At13(+)	783	4	260	29.68	9.05	−0.892
GhGRF5a_Dt	Gohir.D13G196600.1	Dt13(+)	783	4	260	30.03	9.15	−0.957
GhGRF5b_At	Gohir.A10G053300.1	At10(-)	945	4	314	34.88	8.88	−0.694
GhGRF5b_Dt	Gohir.D10G055100.1	Dt10(-)	945	4	314	34.91	8.88	−0.689
GhGRF6a_At	Gohir.A05G033300.1	At05(-)	1,128	4	375	42.73	8.41	−0.955
GhGRF6a_Dt	Gohir.D05G034800.1	Dt05(-)	1,128	4	375	42.73	8.46	−0.955
GhGRF6b_At	Gohir.A13G157400.1	At13(-)	1,005	3	334	36.42	8.30	−0.657
GhGRF6b_Dt	Gohir.D13G162200.1	Dt13(-)	996	3	331	36.13	8.29	−0.667
GhGRF6c_At	Gohir.A02G125800.1	At02(-)	927	4	308	34.63	8.54	−0.777
GhGRF6c_Dt	Gohir.D03G054900.1	Dt03(+)	945	4	314	35.34	8.67	−0.792
GhGRF7_At	Gohir.A12G246800.1	At12(-)	1,491	5	496	53.52	7.25	−0.517
GhGRF7_Dt	Gohir.D12G247500.1	Dt12(-)	1,491	5	496	53.58	6.94	−0.479
GhGRF8_At	Gohir.A12G240900.1	At12(+)	1,398	4	465	50.76	5.89	−0.660
GhGRF8_Dt	Gohir.D12G242200.1	Dt12(+)	1,374	4	457	49.94	5.95	−0.664
GhGRF9a_At	Gohir.A12G223200.1	At12(-)	729	4	242	26.37	9.10	−0.516
GhGRF9a_Dt	Gohir.D12G225800.1	Dt12(-)	729	4	242	26.36	9.30	−0.527
GhGRF9b_At	Gohir.A08G175500.1	At08(-)	498	3	165	17.68	7.68	−0.133
GhGRF9b_Dt	Gohir.D08G194200.1	Dt08(-)	744	3	247	26.71	8.89	−0.470
GhGRF9c_At	Gohir.A11G081800.1	At11(+)	1,437	4	478	52.74	9.65	−0.664
GhGRF9c_Dt	Gohir.D11G086500.1	Dt11(+)	1,335	4	444	48.47	9.54	−0.746

### Phylogenetic analysis and nomenclature of *GRF* genes

**Figure 3:**
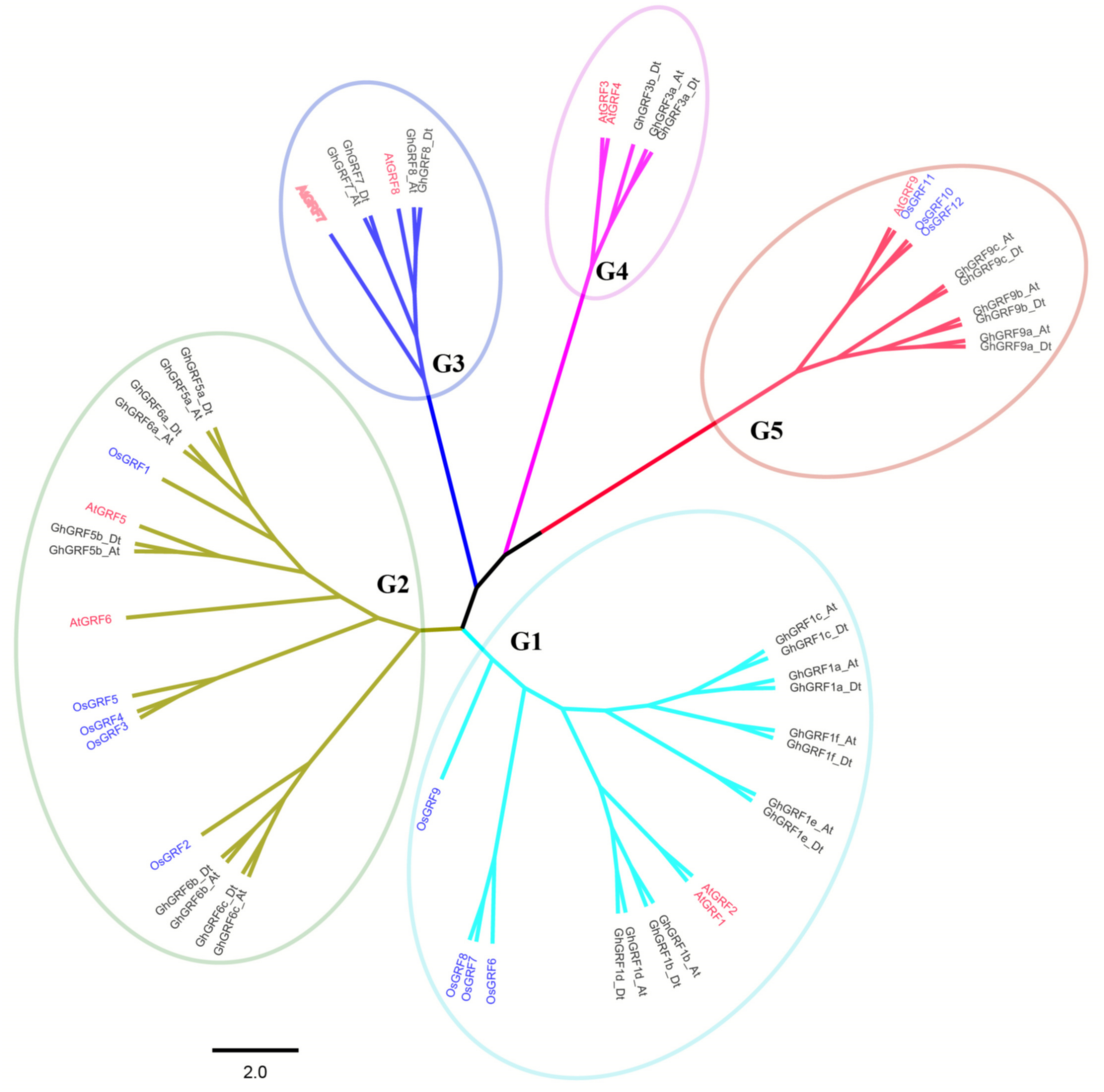
Phylogenetic tree of *GRF* genes. The At and Dt suffixes indicate the A- and D-subgenomes of the upland cotton, respectively. MEGA 5.2 was used for constructing the neighbor joining (NJ) tree. The prefixes Gh, Os, and At stand for *G. hirsutum*, *Oryza sativa*, and *Arabidopsis*, respectively. The scale bar located in the center of the figure represents 2.0 amino acid changes per site.

To better understand the phylogenetic relationships between *GRFs* in *G. hirsutum*, rice, and *Arabidopsis,* a neighbor-joining phylogenetic tree was generated using 56 GRF proteins. *GRF* genes from the three plant species were classified into five groups (G1 to G5) (Fig. 3). To validate the phylogenetic tree constructed using the NJ method, the maximum likelihood (ML) method was used to build another phylogenetic tree. Results indicated that 56 *GRFs* were also divided into five clades based on the ML tree. The topology of the ML tree was slightly different from the NJ tree, but the composition in each clade was the same, indicating that the NJ tree could be used for further analysis.

The number of *GRF* genes differed between the clades of the NJ tree. There were 18, 17, six, five, and 10 members in G1, G2, G3, G4, and G5 clades, respectively. The G1, G2, and G5 groups had the highest coverage of all three species, including the monocot (rice) and dicots (*G. hirsutum* and *Arabidopsis*). In contrast, the G3 and G4 groups contained *GRF* genes only from dicots without no *GRF* member from the monocot. The proportion of *GRF* family members from three different plants in each clade differed. In the G1 group, the number of *AtGRF*, *OsGRF,* and *GhGRF* members were two, four, and 12, respectively, while in the G2 group, the number of *AtGRFs*, *OsGRFs,* and *GhGRFs* were two, five, and ten, respectively. Similarly, in the G3 group, there were two *AtGRFs* and four *GhGRFs* members; the G4 group has two *AtGRFs* and three *GhGRFs* genes. G5 group contained one *AtGRF*, three *OsGRFs,* and six *GhGRFs.* These results indicate that *GRF* families of different plants experienced different evolutionary processes, and that cotton and *Arabidopsis thaliana* are dicotyledons and have a relatively close genetic relationship.

In the phylogenetic tree, names were assigned to each *GhGRF* gene based on their phylogenetic relationship and homologous sequence consistency (Fig. 3). *Gh* is used as a prefix before the *GRF* name of upland cotton, *At* for *Arabidopsis GRFs*. We proceeded according to the following rules of nomenclature: first, *GhGRF* genes were named after their orthologs in *Arabidopsis*, which were part of the same clade of the phylogenetic tree; second, if there were more than one orthologous counterpart of *Arabidopsis*, the *GhGRF* genes were named after the *Arabidopsis* homologous gene possessing the highest sequence consistency with the cotton genes; third, “a”, “b”, and “c” were appended to gene names as suffixes to distinguish the *GRF* genes sharing the common *Arabidopsis* orthologs based on the sequence consistency order from high to low. Finally, At or Dt was attached respectively to the end of each gene name to distinguish the sub-genomes in tetraploid cotton in which the gene was located; “t” means tetraploid.

### Biophysical properties and chromosomal distribution of *GhGRFs*

Physical and biochemical properties include the distribution of genes on the chromosome, gene ID, length of coding sequence (CDS), number of amino acids (protein length), molecular weight (MW), isoelectric point (pI), and grand average of hydropathicity (GRAVY) of *GhGRF* genes and their deduced protein is displayed in Table 1. The CDS length of *GhGRF* genes ranged from 498 bp (*GhGRF9b_At*) to 1,833 bp (*GhGRF1c_At*), and the number of amino acids of their corresponding deduced proteins ranged from 165/17.68 to 610/65.38. Moreover, pI values ranged from 5.89 (*GhGRF8_At*) to 9.65 (*GhGRF9c_At*). In addition, the lowest (−0.957) and highest (−0.373) GRAVY values were computed for the *GhGRF5a_Dt* and *GhGRF1c_Dt,* respectively. We observed that GRAVY scores for all *GhGRF* genes were negative, indicating that all GhGRF proteins were hydrophilic; however, the degree of hydrophilicity varied greatly.

**Figure 4:**
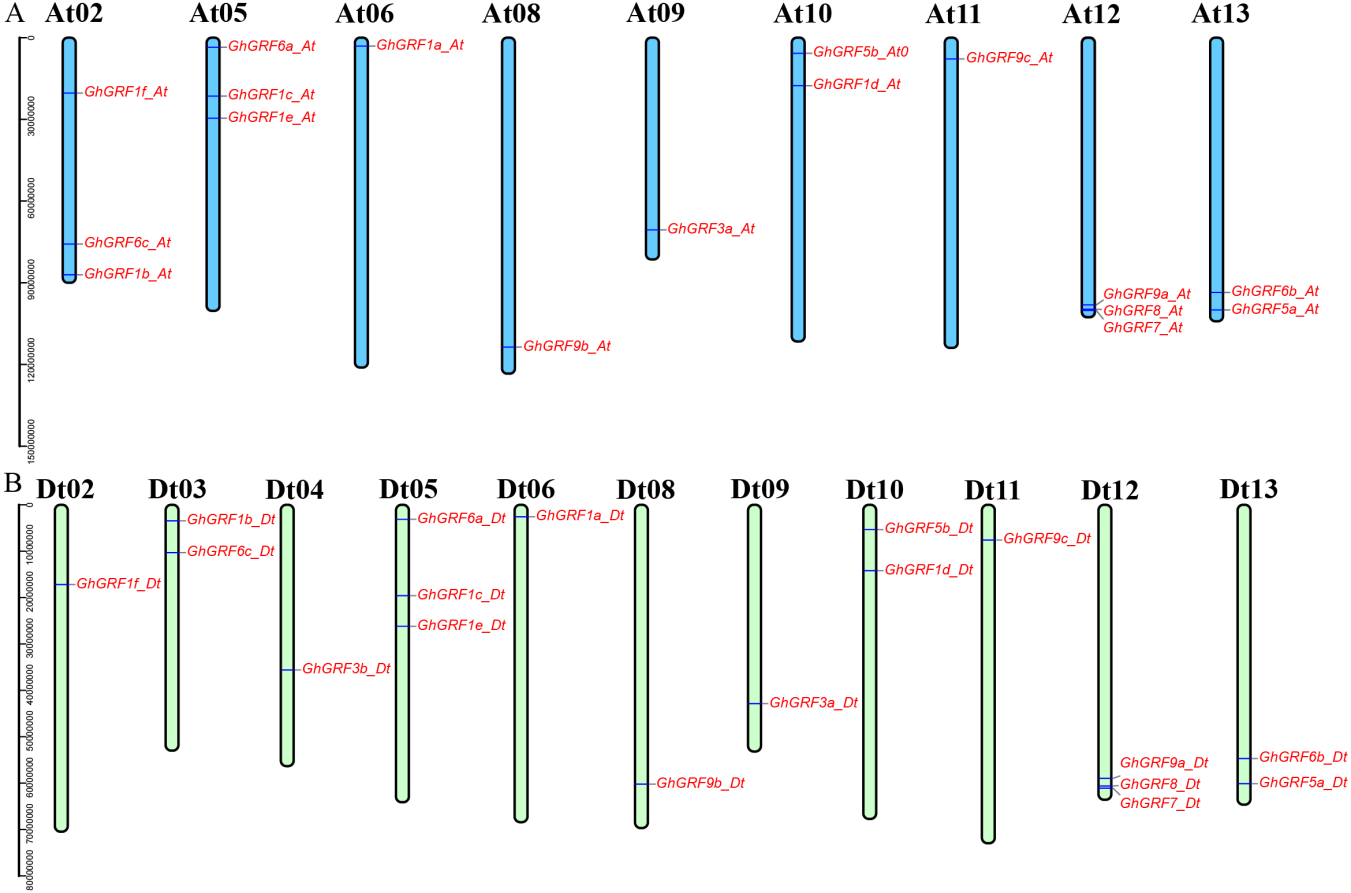
Chromosomal locations of the *GhGRF* genes on the *G. hirsutum* chromosomes. Locations of the *GhGRF* genes on the * G. hirsutum* chromosomes. The blue bars in (A) indicate the A-subgenome chromosome (At), and the yellow green bars in (B) represent the D-subgenome chromosome (Dt). The chromosome number is indicated at the top of each chromosome bar in black. The gene names are labelled in red italics.

Annotation of the *GhGRF* genome sequences (GFF3 profile) helped us to predict the locations of *GhGRF* genes on chromosomes (Fig. 4). The 35 *GRF* genes were unevenly distributed among 20 chromosomes, with 17 genes on nine chromosomes of the At-subgenome and 18 genes on 11 chromosomes of the Dt-subgenome. There were three genes on chromosomes At02, At05, At12, Dt05, and Dt12. For At10, At13, Dt03, Dt10, and Dt13, there were two genes on each chromosome. Ten chromosomes (At06, At08, At09, At11, Dt02, D04, Dt06, Dt108, Dt09, and Dt11) have one *GhGRF* gene. However, six chromosomes (At01, At03-04, At07, Dt01, and Dt07) have no *GhGRF* gene. Four pairs of homologous chromosomes, including At05/Dt05, At06/Dt06, At08/Dt08, and At13/Dt13, have a similar distribution of *GhGRF* genes. However, the gene distribution on three chromosome pairs (At02/Dt02 to At04/Dt04) differed. We inferred that gene loss occurred from the At04 homologous chromosome (*GhGRF3b_Dt)* to the Dt04 chromosome and unidirectional translocation of a chromosome segment containing the two genes, *GhGRF6c-At* and *GhGRF1b_At*, from At03 to At02 occurred (Fig. 4). These results are consistent with those of previous studies (Yang et al., 2017).

### Analysis of gene structure and deduced protein motif

**Figure 5:**
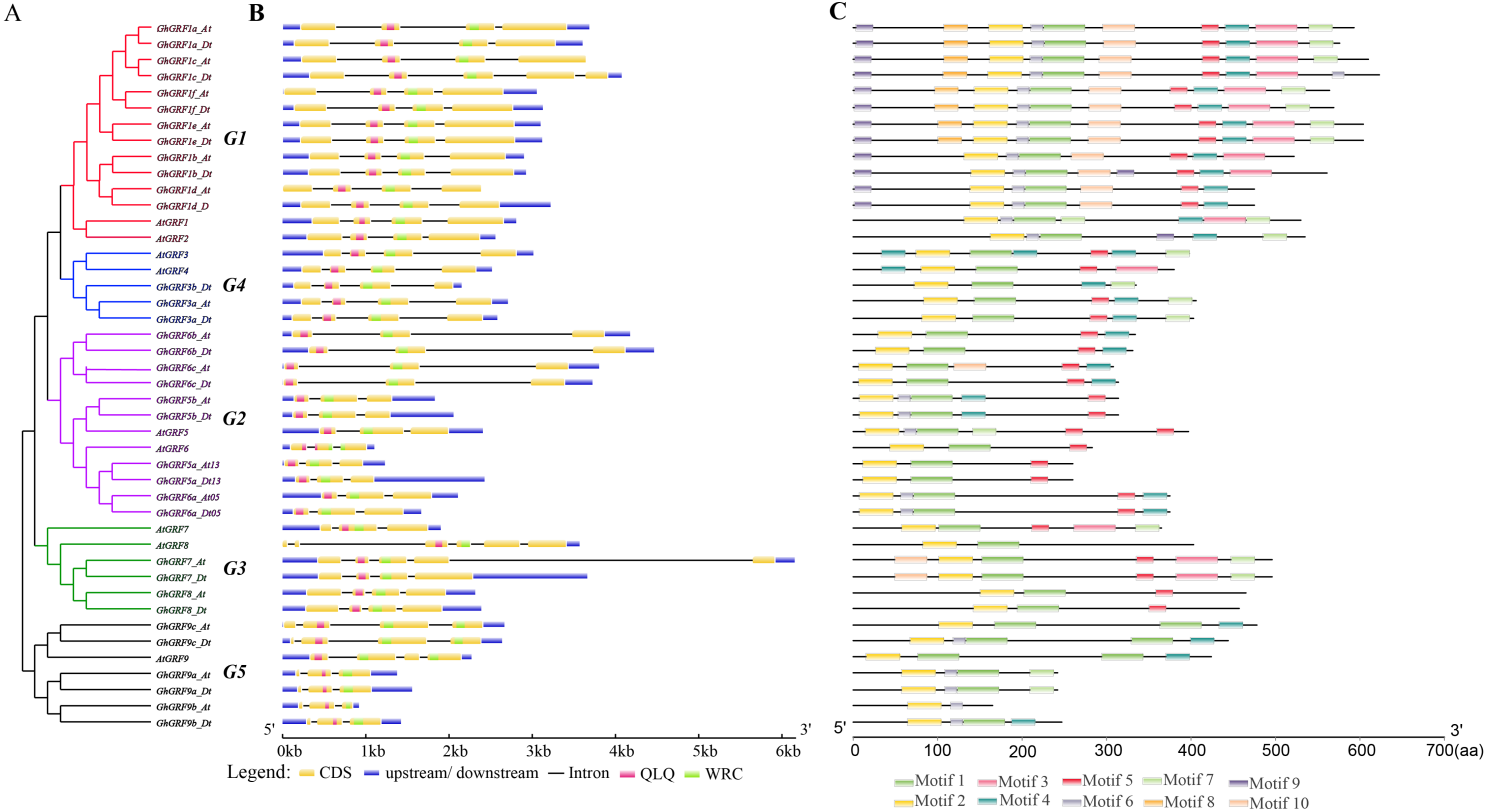
Gene structure and motif analysis of *AtGRFs* and *GhGRFs*. Comparison of the gene structures between *A. thaliana* and *G. hirsutum*. Red, purple, green, blue and black indicate the G1, G2, G3, G4, and G5 clades, respectively. (A) NJ tree of *A. thaliana* and *G. hirsutum*; (B) the number, length, and position of exons and introns within GRF genes; (C) analysis with MEME to investigate 10 conserved motifs of GRF proteins of *Arabidopsis* and *G. hirsutum*.

To explore the exon-intron structure of *GhGRF* genes, their coding and genomic sequences were aligned and the number and positions of exons and introns were detected (Fig. 5B). Results indicated that the structural patterns of genes were conserved between *Arabidopsis* and upland cotton, and the majority of *GhGRF* and *AtGRF* genes possessed 2-3/3-4 introns/exons, respectively. The MEME web server was used to analyze the types and distributions of motifs in *GhGRF* deduced proteins, and ten kinds of motifs were detected (Fig. 5C). Motif 1, characterized by the WRC domain, was predicted in 34 out of 35 *GhGRFs*. Motif 2, characterized by the QLQ domain, was present in all *GhGRFs*. In the C-terminus, there were many conserved motifs, including motifs 3-5, 7, and 10. Moreover, motifs 6, 8, and 9 were primarily located in the N-terminal end close to WRC and QLQ of G1 members. This conserved exon-intron and motif structure pattern supports the evolutionary relationship of *GRF* genes in upland cotton and *Arabidopsis*.

**Figure 6:**
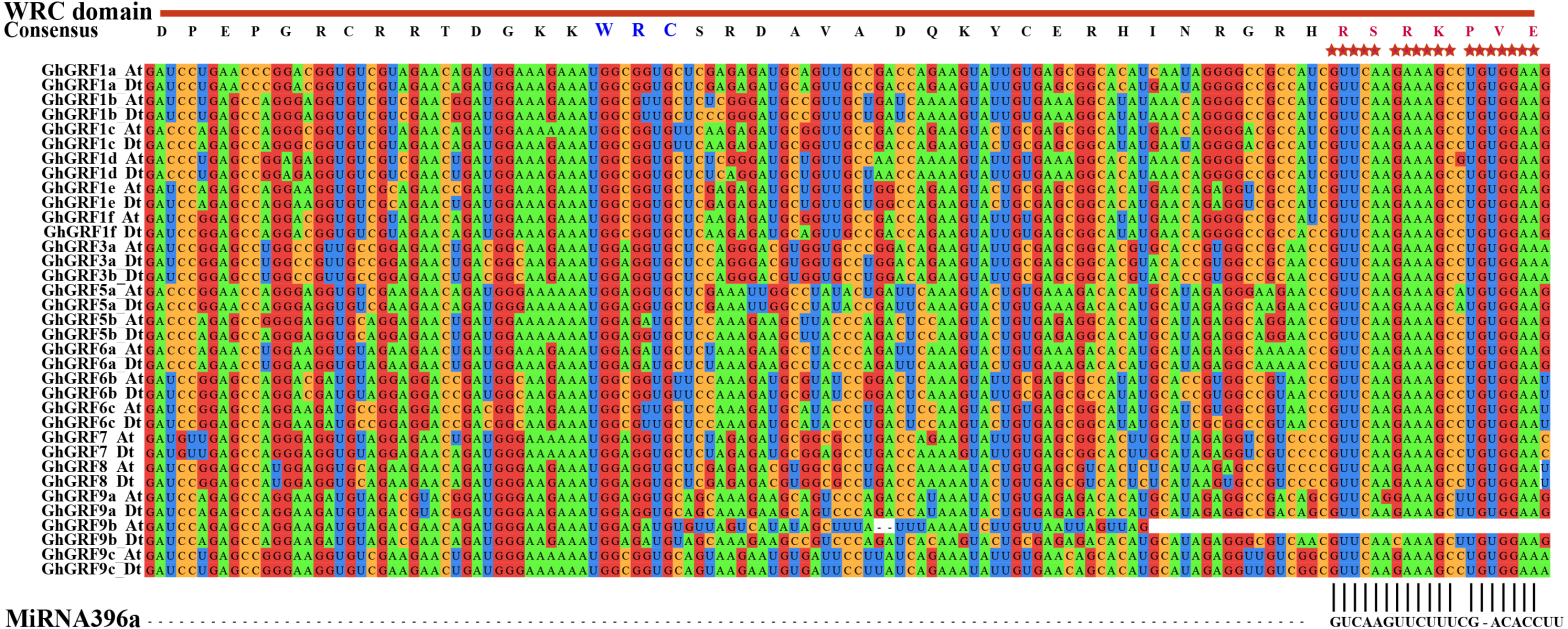
The complementary analysis of the CDS of *GhGRF* genes with MiRNA396a. The red asterisks indicate the conserved reverse complementary sequences of *GhGRF* genes to miRNA396a. The WRC domain consensus sequence of *GhGRF* proteins is at the top of the Figure; the red capital letters indicate the aa fragment corresponding to the conserved RNA sequence fragment of CDS reverse complementary to MiRNA396a.

To investigate whether *microRNA396* inhibits the regulation of *GhGRF* genes on plant growth and development, we tried to find fragments in CDS complementary to *microRNA396a* through multiple sequence alignment using ClustalW in MEGA5.2. Except for *GhGRF9b* (which lost the second half of the gene), all other gene mRNA sequences contained highly conserved fragments reverse complementary to *microRNA396a* (Fig. 6). These results suggested that *microRNA396a* is essential for controlling the function of *GhGRF* genes when regulating plant growth and development by decreasing their transcripts.

### Analysis of *cis*-regulatory elements

To analyze *cis*-regulatory elements, 1,500 bp upstream sequence before the transcriptional start codon was retrieved, and along with the 5′-UTR sequence, was submitted to the PlantCARE database. More than 20 types of *cis*-elements were identified in *GhGRF* promoter regions and divided into four categories based on their functions: (1) Phytohormone responsive elements (ABA, MeJA, auxin, GA, and SA); (2) environmental or stress-responsive elements (light, defense, low-temperature, anaerobic conditions, and drought); (3) *cis*-elements related to growth and development (meristem, palisade mesophyll cells, circadian control, cell cycles, and endosperm); (4) *cis*-elements related to the regulation of bioactive compound metabolism (zein, phytochrome, flavonoid biosynthesis); (Table 2). The distribution of these elements in the promoters regions of *GhGRF* was not uniform, indicating that *GRF* genes became functionally differentiated during germline evolution. The diversity of promoter *cis*-elements demonstrated that the *GRF* genes play an essential role in the growth and development of cotton.

**Table 2:** The *cis*-elements of *GhGRF* promoters.

	I					II								III			IV			V		VI				
	A	B	C	D	E	F	G	H	I	J	K	L	M	N	O	P	Q	R	S	T	U	V	W	X	Y	Z
GhGRF1a_At	1	2	1	0	0	1	15	1	0	0	1	0	2	0	0	0	0	0	0	0	0	1	6	4	1	0
GhGRF1a_Dt	0	1	2	0	0	3	8	0	0	0	0	0	0	0	0	0	0	0	0	0	0	1	8	0	4	0
GhGRF1b_At	1	1	1	1	0	1	11	0	0	0	1	0	0	2	0	0	0	0	0	0	0	1	3	0	5	0
GhGRF1b_Dt	0	0	1	2	0	2	7	0	0	0	1	0	0	1	0	0	0	0	0	0	0	1	4	2	8	0
GhGRF1c_At	4	0	1	0	0	4	12	1	0	0	2	0	2	0	0	0	0	0	0	0	0	0	2	0	2	0
GhGRF1c_Dt	4	0	1	0	0	4	14	1	1	0	0	0	2	0	0	0	0	0	0	0	0	0	3	1	0	0
GhGRF1d_At	2	0	0	0	2	4	10	0	1	1	0	0	0	0	0	0	1	1	0	0	0	0	4	1	2	0
GhGRF1d_Dt	2	1	0	0	2	4	8	0	0	1	0	0	0	0	0	0	0	0	0	0	0	0	4	2	3	0
GhGRF1e_At	2	0	0	2	0	1	9	0	0	0	2	0	2	0	0	0	0	0	0	0	0	1	3	5	3	1
GhGRF1e_Dt	1	0	0	1	0	1	6	1	1	0	1	0	1	0	0	0	0	0	0	0	0	1	2	5	2	0
GhGRF1f_At	2	0	1	1	1	3	12	0	1	0	1	0	2	0	0	0	0	0	0	0	0	0	2	1	1	1
GhGRF1f_Dt	2	0	1	0	0	5	16	0	2	0	1	0	1	0	0	0	0	0	0	0	0	0	2	3	0	0
GhGRF3a_At	4	2	0	0	0	3	10	1	1	2	0	0	3	0	0	0	1	0	0	0	0	0	2	2	0	2
GhGRF3a_Dt	4	3	0	0	0	3	10	2	1	2	0	0	2	0	0	0	0	0	0	0	0	0	2	1	0	2
GhGRF3b_Dt	1	0	0	0	1	3	9	0	2	0	0	0	0	0	0	0	1	0	0	0	0	0	2	3	0	0
GhGRF5a_At	1	1	1	0	1	2	8	0	0	2	0	0	1	0	1	0	0	0	0	0	0	1	9	2	1	0
GhGRF5a_Dt	0	0	2	1	0	5	12	1	0	0	0	0	0	0	0	0	0	0	0	0	0	1	9	2	4	0
GhGRF5b_At	0	0	2	0	0	0	12	1	0	0	1	0	1	0	0	0	0	0	0	0	0	0	4	1	0	0
GhGRF5b_Dt	0	1	2	0	0	4	9	2	0	1	0	0	4	0	0	0	0	0	0	0	0	0	5	2	0	0
GhGRF6a_At	1	1	1	2	1	2	13	0	0	0	1	0	4	0	0	0	2	0	0	1	0	0	11	7	0	0
GhGRF6a_Dt	1	1	1	2	1	1	12	1	0	3	1	0	4	0	0	0	3	0	0	1	0	0	11	4	0	0
GhGRF6b_At	1	0	1	0	1	3	10	0	0	0	0	0	0	1	0	0	1	0	0	0	0	1	4	0	5	0
GhGRF6b_Dt	3	0	2	0	0	2	18	0	1	0	0	1	1	2	0	0	0	0	0	1	0	0	5	3	1	0
GhGRF6c_At	2	0	0	1	0	1	11	1	1	0	0	0	3	1	0	0	1	0	1	1	0	0	4	2	1	0
GhGRF6c_Dt	2	1	0	1	0	0	11	1	1	0	0	0	4	1	0	0	1	0	1	1	0	1	3	4	1	1
GhGRF7_At	1	0	4	1	0	1	12	1	0	0	1	0	2	0	0	1	0	0	0	0	0	0	1	3	4	0
GhGRF7_Dt	4	1	2	1	0	2	9	1	0	0	1	0	2	0	0	2	0	0	0	0	0	0	3	1	6	0
GhGRF8_At	1	1	0	0	0	4	9	0	0	4	0	0	6	1	0	0	0	1	0	0	0	1	9	7	2	0
GhGRF8_Dt	1	1	0	0	0	4	8	0	0	3	0	0	4	1	0	0	0	1	0	0	0	1	8	6	0	0
GhGRF9a_At	5	2	0	0	1	0	9	0	0	1	2	0	0	1	0	0	1	0	0	0	1	1	8	6	1	0
GhGRF9a_Dt	2	0	0	0	0	0	13	0	0	2	2	0	0	2	0	0	2	0	0	0	2	1	12	4	0	0
GhGRF9b_At	1	0	4	0	0	1	13	1	0	0	0	0	1	1	0	0	1	0	0	0	0	1	2	1	3	1
GhGRF9b_Dt	0	0	1	1	0	1	21	0	0	0	0	0	0	0	0	0	1	0	0	0	0	0	3	2	0	0
GhGRF9c_At	4	2	0	2	0	1	7	1	4	0	1	0	1	0	0	0	0	0	0	1	0	0	5	1	1	4
GhGRF9c_Dt	6	2	0	4	0	0	7	0	1	0	1	0	0	0	0	0	0	0	0	0	0	1	9	1	0	0

**Notes.**

Numbers 1, 2, 3…represent the number of repeats of each cis-element whereas 0 indicates absence of the particular cis-element. The capital letters A, B, C…represent various cis elements. More than 20 types of cis-elements were identified and divided into six categories based on their functions. **I**: A, ABE (ACGTG)-abscisic acid responsiveness; B, TGACG-motifs (TGACG)/CGTCA-motifs (CGTCA)-MeJA-responsiveness; C, GARE-motif (TCTGTTG)/P-box (CCTTTTG)-gibberellin-responsive; D, TCA-element (CCATCTTTTT)-salicylic acid responsiveness; E, TGA-element (AACGAC)-auxin-responsive element; **II:** F, ARE (AAACCA)-anaerobic induction; G, AE-box (AGAAACAA)/AT1-motif (AATTATTTTTTATT)/ATC-motif (AGTAATCT)/ATCT-motif (AATCTAATCC)/Box-4 (ATTAAT)/BoxII (TGGTAATAA)/GT1-motif (GGTTAA)/G-Box (CACGTG)/GA-motif (ATAGATAA)/I-box (AGATAAGG, TAGATAACC)/LS7 (CAGATTTATTTTTA)/MRE (AACCTAA)/TCCC-motif (TCTCCCT)/TCT-motif (TCTTAC)-  l ight responsiveness; H, TC-rich repeats (GTTTTCTTAC)-defense and stress responsiveness; I, LTR (CCGAAA)-low-temperature responsiveness; J, MBS (CAACTG)-drought-inducibility; K, W box/WUN-motif (TTGACC, CCATTTCAA, CCACCT); L, AT-rich sequence (TAAAATACT)-maximal elicitor-mediated activation; M, STRE, (AGGGG); **III:** N, O2-site [GATGA(C/T)(A/G)TG(A/G)]-Zein metabolism regulation; O, Unnamed-1(GGATTTTACAGT)-phytochrome down-regulation expression; P, MBSI (TTTTTACGGTTA)-flavonoid biosynthetic genes regulation; **IV”** Q, CAT-box (GCCACT)-meristem expression; R, HD-Zip 1 [CAAT(A/T)ATTG]-differentiation of the palisade mesophyll cells; S, GCN4_motif (TGAGTCA)-endosperm expression; **V:** T, Circadian (CAAAGATATC)-circadian control; U, MSA-like [(T/C)C(T/C)AACGG(T/C)(T/C)A]-cell cycle regulation; **VI:** V, WRKY binding site, WRE, (CAACTG)-cis-element for proteins; W, MYB binding site, (TAACCA, CAACAG, CAACCA, TAACTG); X. MYC binding site, (CAATTG, CATGTG, CATTTG, TCTCTTA); Y, ERF binding site; ERE, (ATTTTAAA, ATTTCATA); Z, DREB binding site.

### Expression patterns of *GhGRF* genes

**Figure 7:**
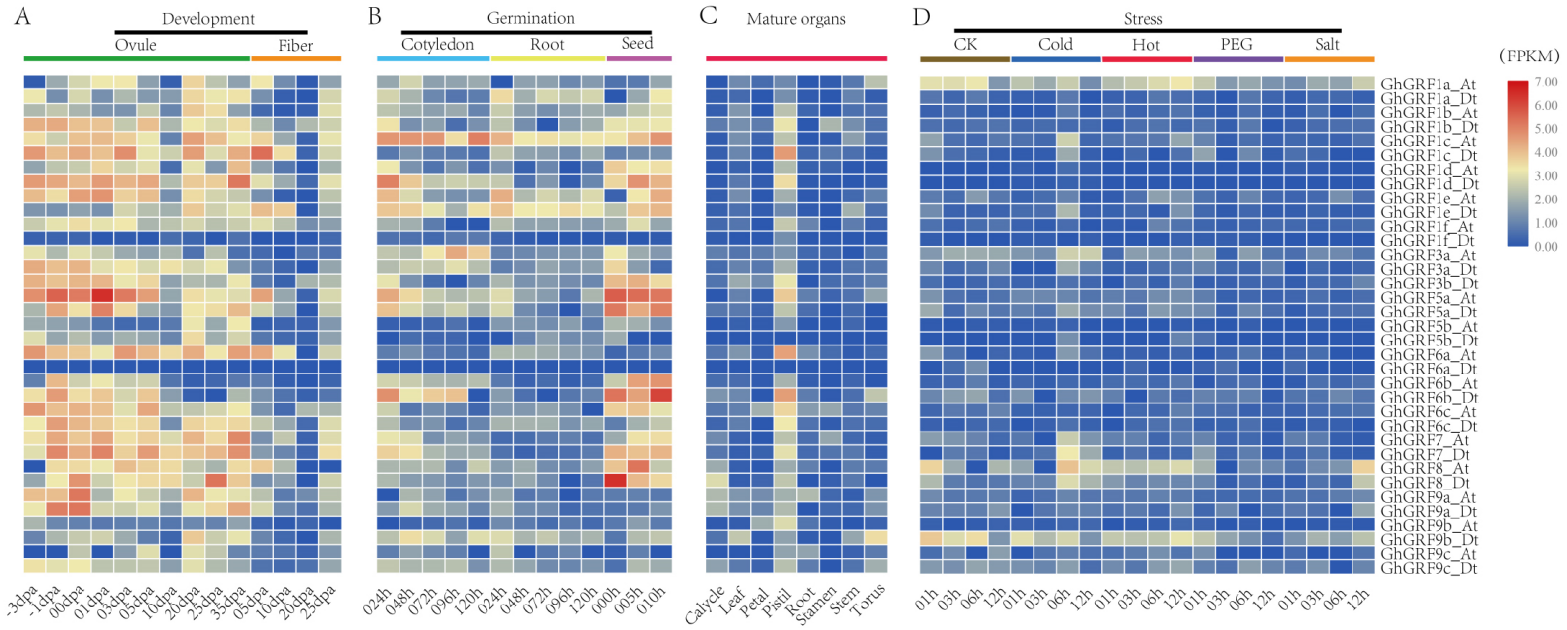
Expression analysis of *GhGRF* genes in *G. hirsutum* TM-1. (A) Samples from developing ovule and fiber; (B) samples from seed germination and cotyledon, root growth; (C) samples from mature organs including of calycle, leaf, petal, pistil, root, stamen, stem, and torus; (D) samples under stress including cold, heat, PEG and salt. The white numbers in boxes are the original data. od: ovule development; fd: fiber development; cg: cotyledon growth; rg: root growth; sg: seed germination; dpa: day post anthesis; the minus sign (-) in front of dpa means before anthesis; PEG: polyethylene glycol.

To analyze the expression patterns of *GhGRF* genes, publicly available transcriptomic data was retrieved and used to describe the expression levels of *GhGRF* genes in mature organs, developing tissues, and abiotic stress conditions (Fig. 7). The expression patterns of *GhGRF* genes in different tissues indicated that most *GhGRF* genes transcripts developed high amounts of ovules and fibers, except for two genes (*GhGRF1f_Dt* and *GhGRF6a_Dt*) whose expression was barely detected during ovule and fiber development (Fig. 7A). More than half of the *GhGRF* genes were highly expressed during seed germination and the root and cotyledon development processes (Fig. 7B). As shown in Fig. 7C, 27 genes exhibited high expression in mature organs (pistil). The transcript level of 2–5 genes was high for other organs such as calycle, leaf, petal, root, stamen, stem, and torus. Only one gene (*GhGRF9b_Dt*) indicated a high transcript level in the torus.

We next observed the expression levels of *GhGRF* genes against abiotic stresses (cold, heat, salt, and PEG) using transcriptomic data. *GhGRF1a_At* and *GhGRF9b_Dt* exhibited downregulated expression under the exposure of cold and PEG, while the expression levels of these two genes were upregulated under heat and salt stress. Moreover, the expression of *GhGRF1c _At* was high at 6 h and 12 h of cold and heat treatment, while *GhGRF3a_At* was up-regulated only in response to cold. However, *GhGRF8_At* was down-regulated in response to cold, heat, and salt.

**Figure 8:**
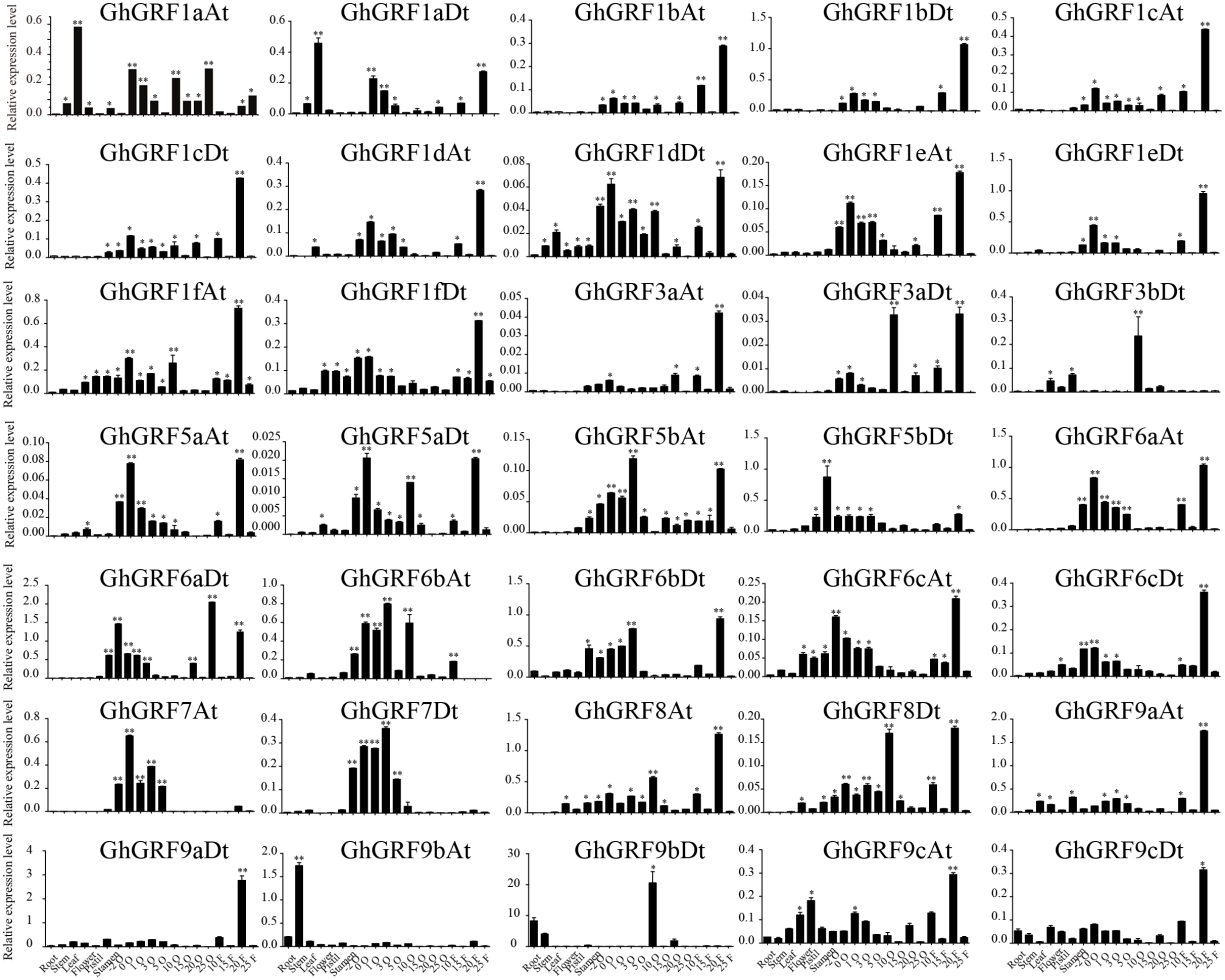
Expression pattern analysis of 35 *GhGRF* genes in different cotton tissues. Error bars indicated the standard deviations (SD) among three independent biological repeats. Differences between groups were compared using the *t*-test (* *p* < 0.05, ** *p* < 0.01).

To validate these results, we performed RT-qPCR analysis of 35 *GhGRF* genes using cotton tissues (root, stem, leaf, flower, pistil, stamen, ovule (−2, 0, 1, 3, 5, 10, 15, 20, 25), fiber (10, 15, 20, 25), abiotic stress (NaCl and PEG), and hormonal treatments (IAA, BL, and GA). Interestingly, tissue-specific expression patterns were similar to the previously published RNA - seq data. Most *GhGRF* genes (*GhGRF1aAt, GhGRF1aDt GhGRF1bAt, GhGRF1bDt, GhGRF1cAt, GhGRF1cDt, GhGRF1dAt, GhGRF1dDt, GhGRF1eAt, GhGRF1eDt, GhGRF1fAt, GhGRF1fDt, GhGRF3aAt, GhGRF3aDt, GhGRF5aAt, GhGRF5aDt, GhGRF5bAt, GhGRF5bDt, GhGRF6aAt, GhGRF6aDt, GhGRF6cAt, GhGRF6cDt, GhGRF8At, GhGRF8Dt, GhGRF9cAt,* and *GhGRF9cDt*) exhibited preferentially high expression during ovule and fiber development, indicating that they are potentially involved in ovule and fiber development (Fig. 8). The tissue expression trend of most genes is consistent when qRTPCR with transcriptome data, including *GhGRF1dDt, GhGRF1eAt, GhGRF5aAt, GhGRF5aDt, GhGRF6aDt, GhGRF6bAt, GhGRF7At, and GhGRF7Dt.*

**Figure 9:**
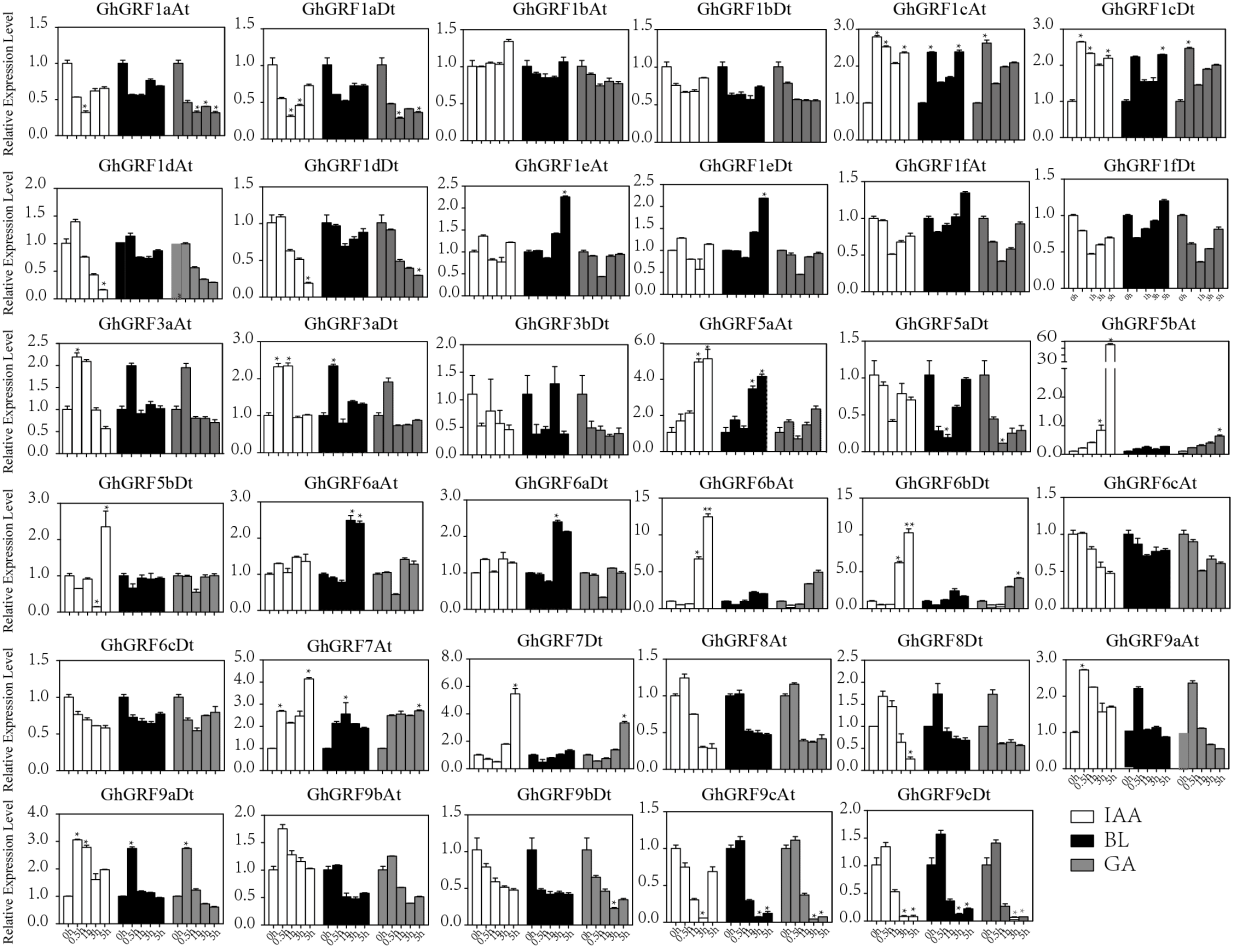
Expression analysis of *GhGRF* genes for two abiotic stresse. Relative expression patterns of *GhGRF* genes with five hormonal stresses (BL, GA and IAA) at different time points. 0 h represent CK. Differences between groups were compared using the *t*-test (* *p* < 0.05, ** *p* < 0.01).

**Figure 10:**
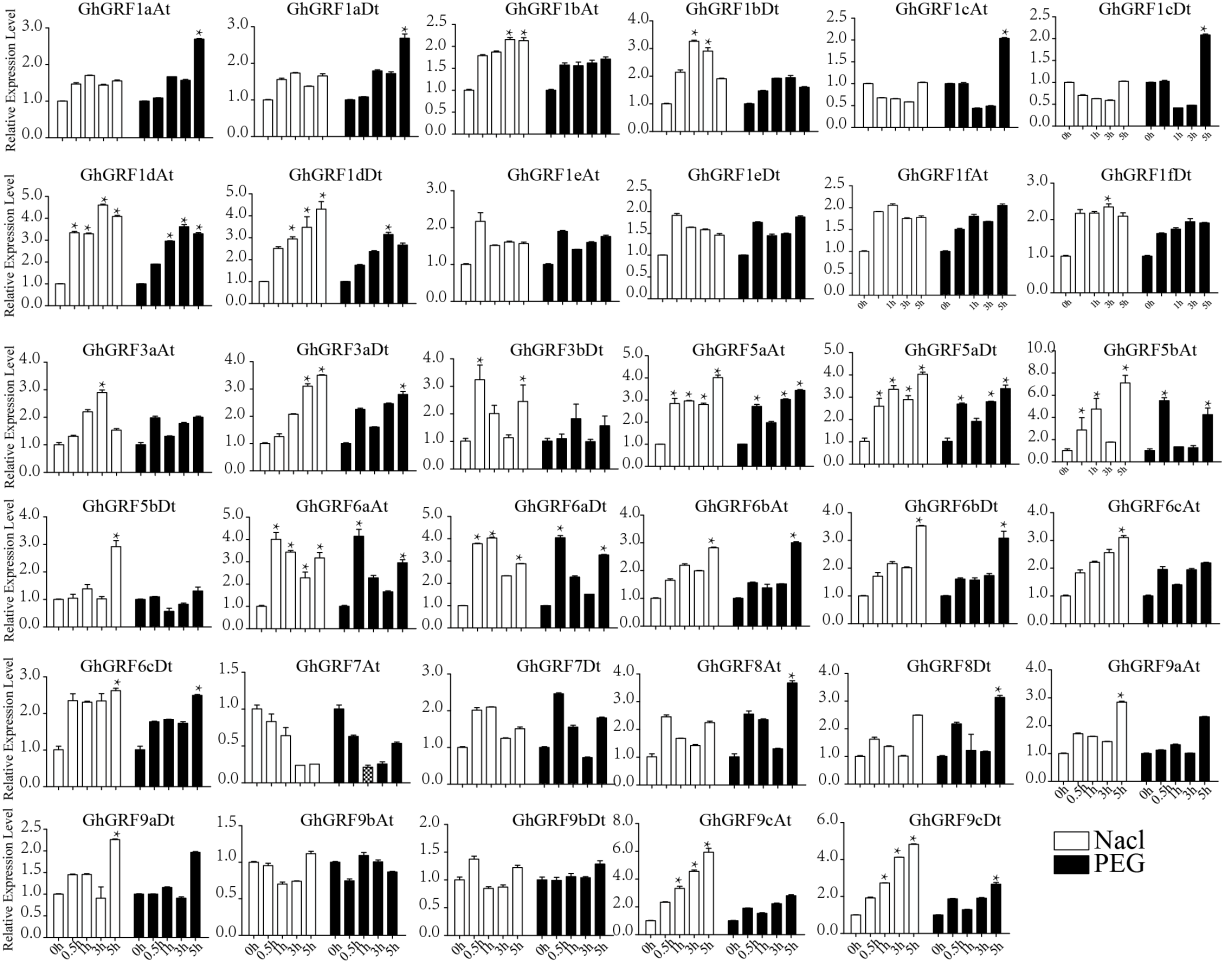
Relative expression patterns of *GhGRF* genes with five hormonal stresses. Expression analysis of *GhGRF* genes for two abiotic stresses including NaCl and PEG. Error bars exhibits standard deviations (SD) among three independent biological repeats. 0 h represent CK. Differences between groups were compared using the *t*-test (* *p* < 0.05, ** *p* < 0.01).

Expression pattern analysis for abiotic and hormonal stresses indicated that all *GhGRF* experienced both up- and down-regulated expression at different time points. The expression level of *GhGRF1aAt, GhGRF1aDt, GhGRF1bAt, GhGRF1bDt, GhGRF1dAt, GhGRF1dDt, GhGRF1eAt, GhGRF1eDt, GhGRF1fAt, GhGRF1fDt, GhGRF3aAt, GhGRF3a Dt, GhGRF3bDt, GhGRF5aAt, GhGRF5aDt, GhGRF5bAt, GhGRF6aAt, GhGRF6aDt, GhGRF6bAt, GhGRF6bDt, GhGRF6cAt, GhGRF6cDt, GhGRF8At, GhGRF9aDt, GhGRF9cAt, GhGRF9cDt* was significantly higher for all time points under all abiotic stresses (Fig. 9), suggesting that these *GhGRF* genes could improve plant resistance to abiotic stresses and are potential candidate genes for further study. The transcript level of *GhGRF7At* decreased at all time points with NaCl and PEG treatment compared to the control. The transcription level of other genes decreased at 0.5, 1, and 3 h after NaCl and PEG treatment, including *GhGRF1cAt* and *GhGRF1cDt*. For hormonal treatments, including IAA, BL, and GA, some genes (*GhGRF1cAt, GhGRF1cDt, GhGRF5aAt*) had relatively high expression levels at all time points. This highlights the important role they play in hormone signaling pathways (Fig. 10). Other *GhGRF* genes exhibited moderate to low expression when exposed to IAA, BL, and GA treatments.

## Discussion

*GRF* family transcription factors are involved in regulating various life processes during plant growth and development by controlling cell division (Horiguchi, Kim & Tsukaya, 2005; Kim & Kende, 2004; Kim & Lee, 2006; Lee et al., 2009; Omidbakhshfard et al., 2018), providing an opportunity for genetically improving crops including rice, wheat, and *Brassica napus* (Che et al., 2015; Chen et al., 2019; Duan et al., 2015; Hu et al., 2015; Li et al., 2016; Liu et al., 2012; Raz et al., 2018; Sun et al., 2016). In our study, 35 *GRF* genes were identified in *G. hirsutum*, which was the highest number of *GRF* of all selected species (Cao et al., 2016; Choi, Kim & Kende, 2004; Fi̇li̇z, Koc & Tombuloğlu, 2014; Kim, Choi & Kende, 2003; Wang et al., 2014a; Zhang et al., 2008; Zhang et al., 2018). We primarily analyzed *GhGRFs* and identified their potential functions in growth and development.

Previous studies reported that GRF proteins with regulatory functions in plant growth and development share common structural features that are characterized by two highly conserved motifs, QLQ and WRC (Choi, Kim & Kende, 2004; Kim, Choi & Kende, 2003; Kim & Kende, 2004; Van der Knaap, Kim & Kende, 2000). In this study, we performed a comparative analysis of the structural features of *GhGRFs* and *AtGRFs* and found that they have very conserved QLQ and WRC domains, including TQL (motif 4), GGPL (motif 3), and FDW (motif 5). Only one gene, *GhGRF9b_At*, was shortened without the end-half of the WRC domain and the C-terminus. Therefore, most *GhGRFs* regulate the expression of downstream genes involved in cell division and therefore control cotton growth and development.

Previous studies reported that *GRF* family TFs were highly expressed in growing and developing tissues, including shoot tips, flower buds, and roots, but that they respond weakly in mature stem and leaf tissues (Kim, Choi & Kende, 2003). We found that nearly all *GhGRF* genes were highly expressed during the ovule development stages, and nearly half of the selected genes were highly expressed during the development of fiber tissues. The expression level of most *GhGRF* genes was up-regulated under stress treatment (PEG and NaCl) and hormonal treatment (IAA, BL, and JA). These results suggest a possible role for *GhGRF* genes in regulating the growth and development of cotton plants by cell division in young and growing tissues.

It is commonly believed that gene expression is controlled by promoter regions, which function as a multi-channel switch. Our analysis of promoter *cis*-elements indicated that the *GhGRF* gene promoter regions contain various *cis*-elements related to light, low temperature, drought, biological stresses, and a variety of hormones, including auxin, GA, SA, MeJA, and ABA binding with TFs such as ERF, MYB, MYC, and DREB. Our research demonstrated that these *GhGRF* genes could be essential for signal transduction pathways and integrating the internal and external environment. Consistent with previous studies of *AtGRFs*, we found that they were greatly accumulated in *GhGRF* gene transcripts and less so in mature tissues. It has been documented that *microRNA396* is the major repressor of *GRF* gene expression (Baucher et al., 2013; Bazin et al., 2013; Beltramino et al., 2021; Chen, Luan & Zhai, 2015; Debernardi et al., 2014; Debernardi et al., 2012; Hewezi et al., 2012; Liu et al., 2009; Liu et al., 2014; Rodriguez et al., 2010; Yang et al., 2009). In our study, we found that a reverse complementary sequence of *microRNA396a* existed in all *GRF* gene CDS except *GhGRF9b_At*, suggesting that *microRNA396a* might be the reason for the lower expression level of *GhGRF* genes in mature organs.

Our results indicate that *GhGRF* genes can be used in cotton genetic engineering to improve fiber and cottonseed oil yield. However, little is known about the regulatory network and mechanism of *GhGRF* genes, ecological risks and technological limitations have hampered further study. However, progress has been made researching the function and mechanism of *OsGRF4* with mutations in *GL2* (Che et al., 2015), *GS2* (Duan et al., 2015; Hu et al., 2015), *GLW2* (Li et al., 2016), *PT2* (Sun et al., 2016), *LGS1* (Chen et al., 2019), and *TtGRF4-A* (Raz et al., 2018) (homologs to *OsGRF4* in wheat). These spontaneous mutations of *GRF* genes occurred in regions reverse complementary to *microRNA396* and stopped the *microRNA396* inhibition of *GRF* genes, leading to high accumulation of *GRF* mRNA. However, these mutations did not change the structure and function of proteins encoded by the *GRF* genes, meaning that a high accumulation of *GRF* mRNA promotes extra growth and development. For example, Large grain Size 1 (*LGS1*) is an allele of the *OsGRF4* gene that contains 2 bp missense mutation in the *OsGRF* gene coding region reverse complementary to *microRNA396*, with no influence on its normal function. However, the mutation disrupts the pairing of the *LGS1* mRNA with *microR396,* leading to the up-regulation of the *OsGRF4* transcript accumulation, the promotion of plant growth and development, the formation of larger grains, and increased cold tolerance (Chen et al., 2019). However, the development of the CRISPR/Cas9 system has made it possible to perform targeted mutagenesis on functional genes in plants (Belhaj et al., 2015; Demirci, Zhang & Unver, 2018; Samanta, Dey & Gayen, 2016).

Inspired by this research progress, we proposed a feasible technical route for the molecular improvement of cotton production. The natural mutations mentioned above can be mimicked using gene-editing techniques to produce desirable mutations of some *GhGRF* genes with high frequency. Specifically, the CRISPR/Cas9 system can be used to edit the bases in reverse complementary fragments of the *GhGRF* genes to the *microRNA396a* sequences and produce gene mutations, making its transcripts unable to pair with *microRNA396a* without damaging its ability to translate into proteins with normal function. Transgenic ingredients of the Cas9 vector can be removed through hybridization and selection. Using this method, we can easily obtain mutants possessing expected agronomic traits, which can be used in cotton breeding and production, avoiding the negative effects of these transgenic technologies.

## Conclusions

In this study, 35 *GRF* genes were identified in *G. hirsutum*, with highly conserved deduced protein structures. *GhGRFs* shared the QLQ and WRC domains at the N-terminal end with *GRFs* in *Arabidopsis* and rice, which laid the structural foundation for the regulation of its growth and development. The phylogenetic tree revealed the homologous relationship among *GRF* family members from *Arabidopsis*, *G. hirsutum,* and rice, indicating that the *GhGRF* genes have similar functions and mechanisms as *Arabidopsis* rice. The mRNA sequences of *GRF* genes contain reverse complementary sequences with *microRNA396a*, indicating that *microRNA396a* can effectively inhibit its function. We found that the expression of most *GhGRF* genes was high during the growth and development of ovules, fibers, and pistils. The significant changes in the expression levels of *GhGRF* genes under stress further illustrate that these genes are required for growth and development in normal environments and under stress conditions. Promoter *cis*-elements and expression patterns analyses indicated that the *GhGRF* genes play a key role in regulating plant growth and development. Therefore, the production of oil and fiber can be increased using CRISPR/Cas9 technology to conduct targeted mutagenesis in the reverse complementary fragment of *GhGRF* genes with microRNA396a to promote the growth and development of cotton.

##  Supplemental Information

10.7717/peerj.13372/supp-1 Supplemental Information 1 He domain analyses of 42 *GhGRF* candidates identified from HAU The domain analyses of 42 *GhGRF* candidates identified from HAU; 33 out of 42 protein sequences were confirmed to contain the QLQ domain adjacent to the WRC domain in the N-terminal end. The red font with preceding asterisks indicates discarded sequences.

10.7717/peerj.13372/supp-2 Supplemental Information 2 The domain analyses of 41 *GhGRF* candidates identified from ZJU The domain analyses of 41 *GhGRF* candidates identified from ZJU; 35 out of 41 protein sequences were confirmed to contain the QLQ domain adjacent to the WRC domain in the N-terminal end. The red font with preceding asterisks indicated discarded sequences

10.7717/peerj.13372/supp-3 Supplemental Information 3 Maximum likelihood phylogenetic tree of *GRF* genes Maximum likelihood phylogenetic tree of *GRF* genes indicating that *GRF* genes could be divided into five clades including G1, G2, G3, G4, and G5. MEGA 5.2 was used for constructing the maximum likelihood (ML) tree. The prefixes Gh, Os, and At stand for *G. hirsutum*, *Oryza sativa*, and *Arabidopsis thaliana*, respectively. The At and Dt suffixes indicate the A- and D-subgenomes of the upland cotton, respectively. The scale bar located in the center of figure represents 2.0 amino acid changes per site.

10.7717/peerj.13372/supp-4 Supplemental Information 4 The conservative sequence LOGOs of ten motifs analyzed using MEME

10.7717/peerj.13372/supp-5 Supplemental Information 5 Quantitative raw data of tissue expression of GhGRF gene family

10.7717/peerj.13372/supp-6 Supplemental Information 6 Raw data of hormone processing response of GhGRF gene at different time points

10.7717/peerj.13372/supp-7 Supplemental Information 7 Raw data of GhGRF gene response after NaCl and PEG treatment at different time points

## Additional Information and Declarations

### Data Availability

The following information was supplied regarding data availability: The raw data are available in the Supplemental Files.
