# Supplementary figures and images for: Genome-wide identification and expression profile of GhGRF gene family in Gossypium hirsutum L
Source: PeerJ. 2022 May 13;10:e13372. doi: 10.7717/peerj.13372 (PMC9109687; doi:10.7717/peerj.13372)

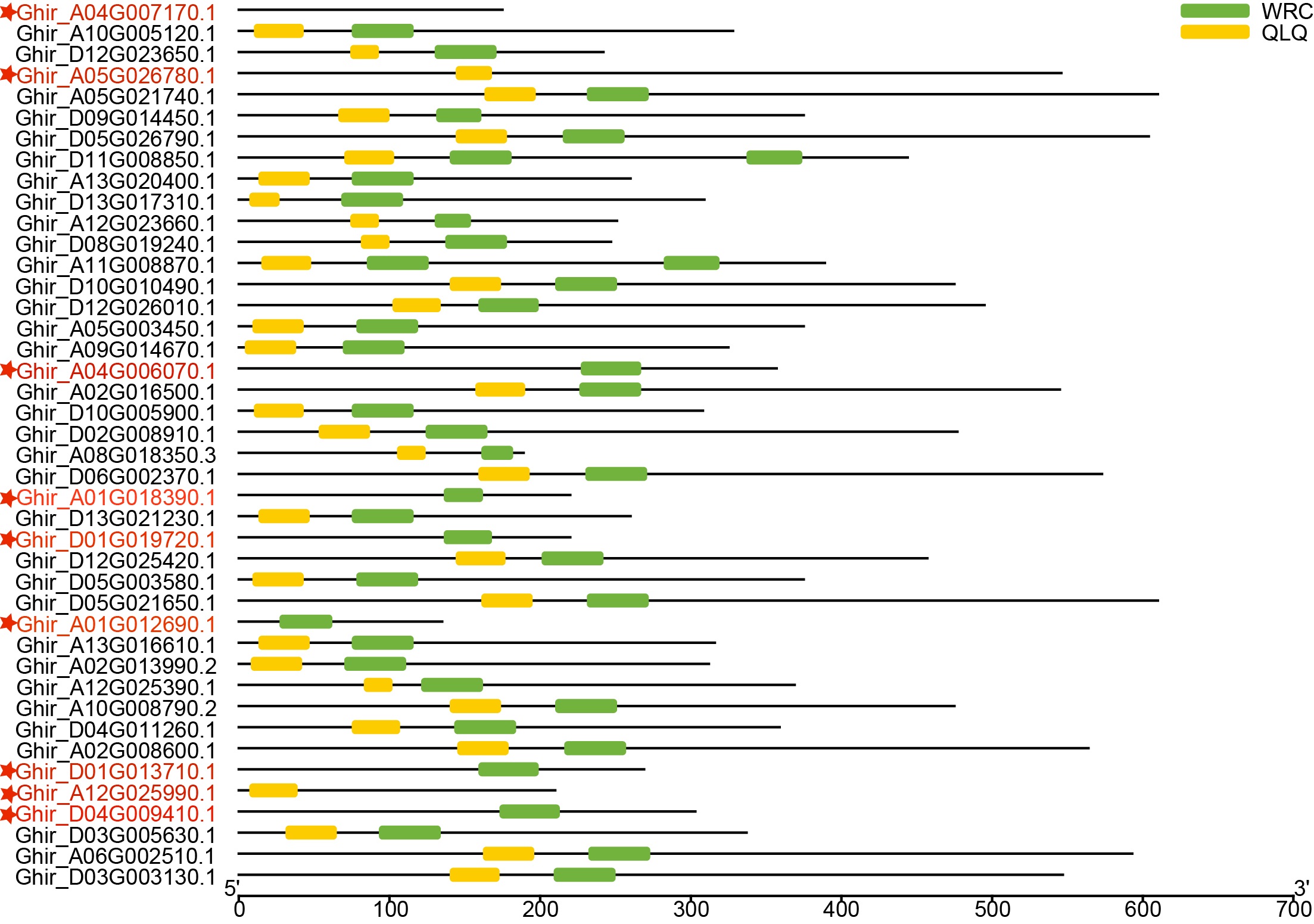

Supplement: Supplemental Information 1 — The domain analyses of 42 GhGRF candidates identified from HAU; 33 out of 42 protein sequences were confirmed to contain the QLQ domain adjacent to the WRC domain in the N-terminal end. The red font with preceding asterisks indicates discarded sequences. [file peerj-10-13372-s001.jpg]

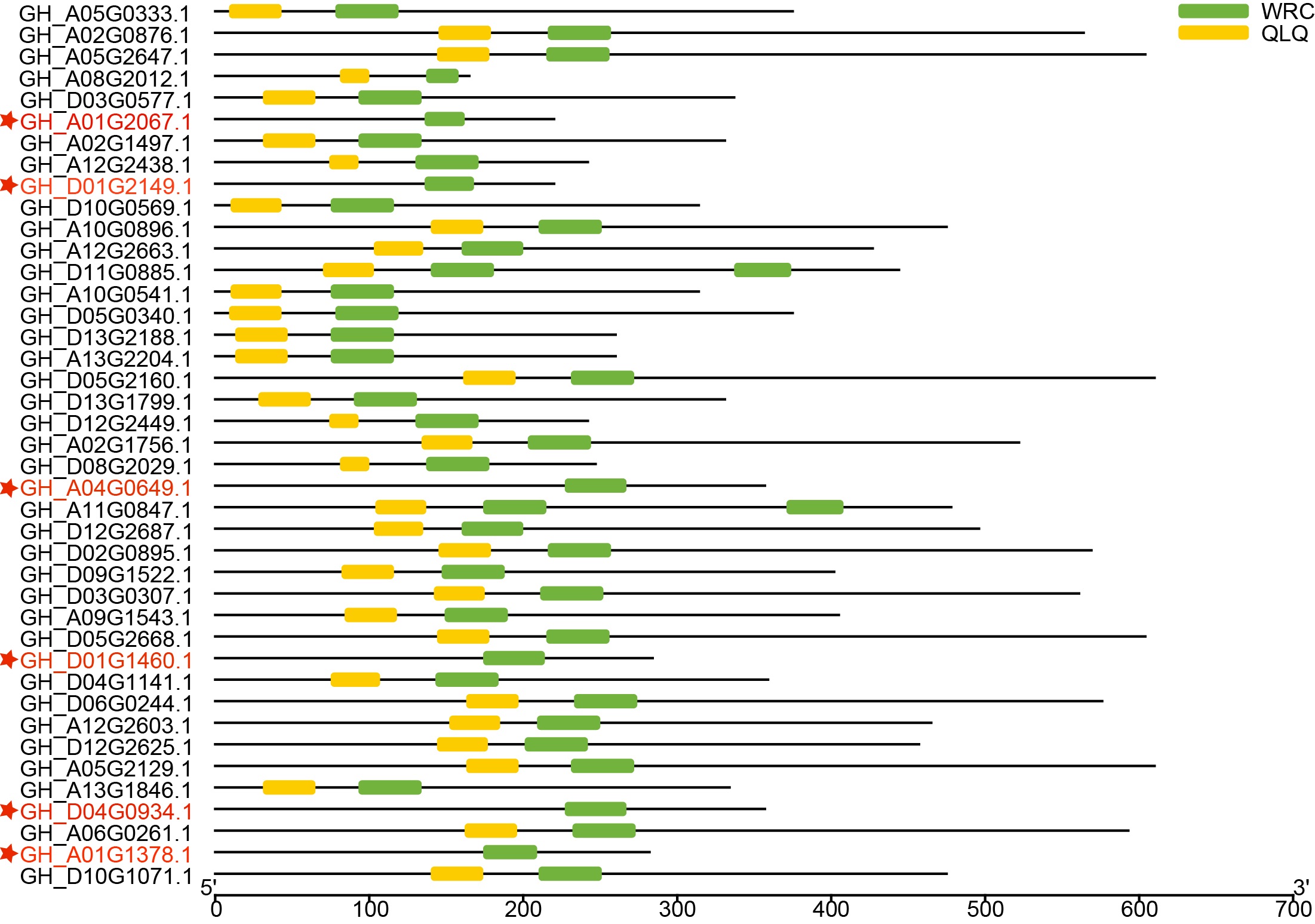

Supplement: Supplemental Information 2 — The domain analyses of 41 GhGRF candidates identified from ZJU; 35 out of 41 protein sequences were confirmed to contain the QLQ domain adjacent to the WRC domain in the N-terminal end. The red font with preceding asterisks indicated discarded sequences [file peerj-10-13372-s002.jpg]

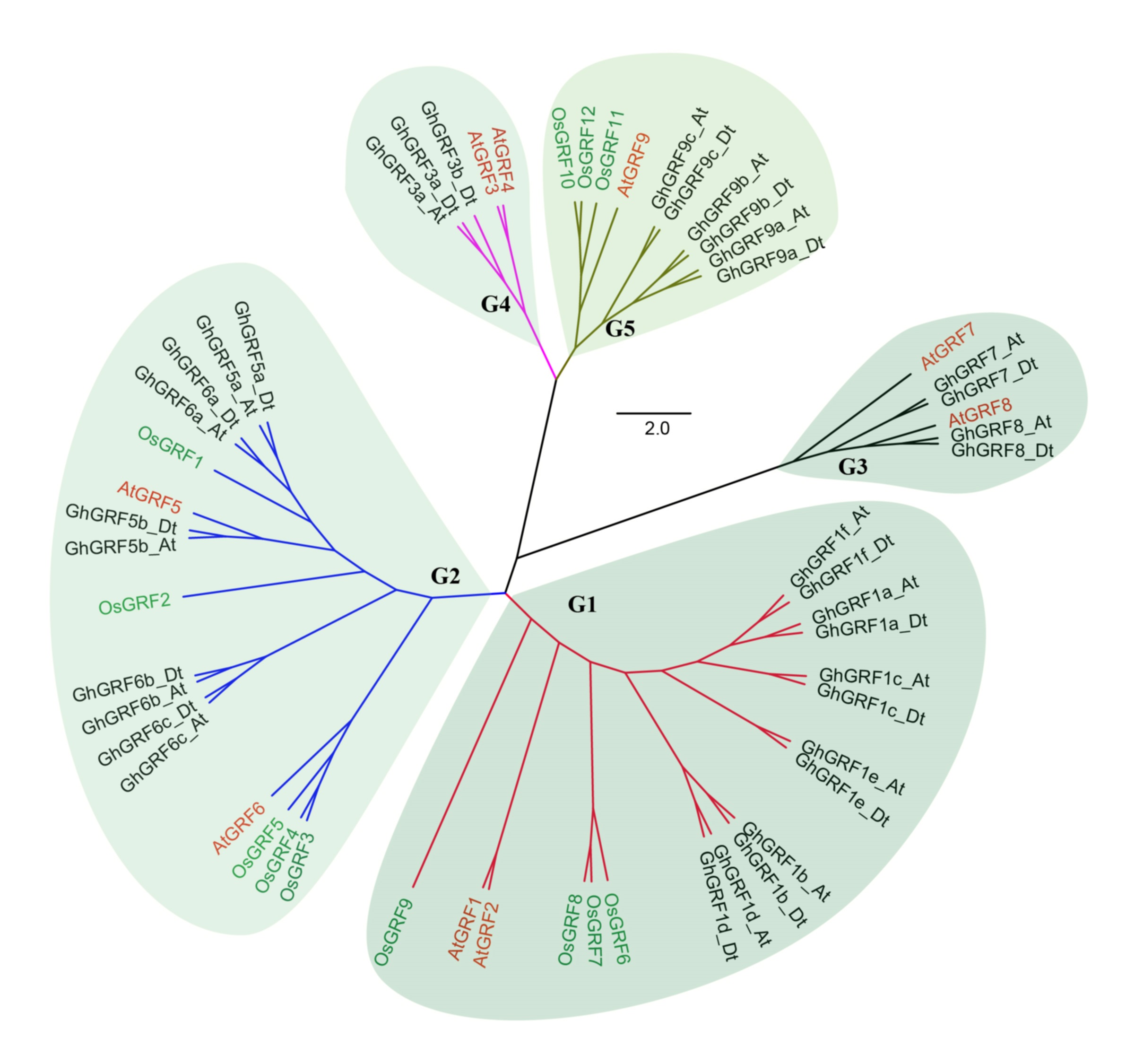

Supplement: Supplemental Information 3 — Maximum likelihood phylogenetic tree of GRF genes indicating that GRF genes could be divided into five clades including G1, G2, G3, G4, and G5. MEGA 5.2 was used for constructing the maximum likelihood (ML) tree. The prefixes Gh, Os, and At stand for G. hirsutum, Oryza sativa, and Arabidopsis thaliana, respectively. The At and Dt suffixes indicate the A- and D-subgenomes of the upland cotton, respectively. The scale bar located in the center of figure represents 2.0 amino acid changes per site. [file peerj-10-13372-s003.jpg]

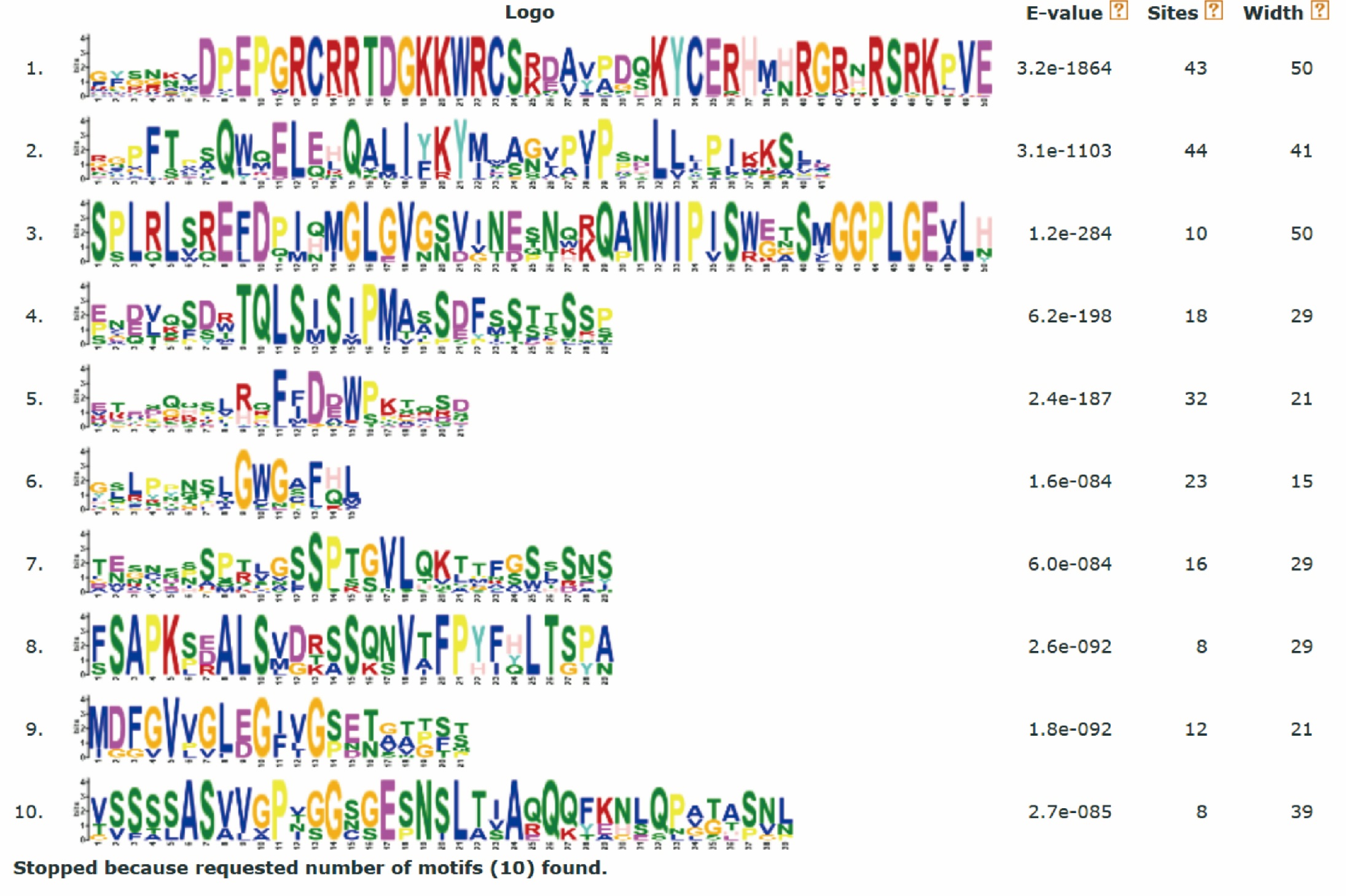

Supplement: Supplemental Information 4 [file peerj-10-13372-s004.jpg]
